# Hydrocephalus management pathways in pediatric medulloblastoma: a retrospective cohort study

**DOI:** 10.3389/fonc.2026.1928797

**Published:** 2026-09-08

**Authors:** Yunwei Ou, Yiqiao Xu, Jingzhe Yuan, Haoqi Zeng, Chunde Li, Jian Gong

**Affiliations:** 1 Department of Neurosurgery, Beijing Tiantan Hospital, Capital Medical University, Beijing, China; 2 The Institute of Artificial Intelligence, Hefei Comprehensive National Science Center, Hefei, Anhui, China

**Keywords:** endoscopic third ventriculostomy, hydrocephalus, internal evaluation, medulloblastoma, propensity score matching, ventriculoperitoneal shunt

## Abstract

**Objective:**

To compare recorded postoperative intracranial infection and recurrent hydrocephalus across three hydrocephalus management pathways in children with medulloblastoma and to conduct an exploratory internal prediction analysis for postoperative infection after ventriculoperitoneal (VP) shunting.

**Methods:**

This retrospective single-center cohort and prediction-model study included 420 children with medulloblastoma and preoperative hydrocephalus treated at Beijing Tiantan Hospital from January 1, 2014, through March 1, 2018. Patients were grouped by initial definitive hydrocephalus pathway: preoperative VP shunt, upfront tumor resection without permanent preoperative diversion, or preoperative endoscopic third ventriculostomy (ETV). Pairwise propensity score matching was performed for VP shunt versus upfront resection, ETV versus upfront resection, and ETV versus VP shunt. The minimum planned postoperative follow-up was 6 months. Candidate VP-shunt infection models were evaluated by repeated stratified 5-fold cross-validation, with imputation, standardization, and sampling fitted within training folds only.

**Results:**

The cohort included 227 VP-shunt, 140 upfront-resection, and 53 ETV patients. Postoperative infection occurred in 55/227 patients (24.2%) after VP shunting, 31/140 (22.1%) after upfront resection, and 5/53 (9.4%) after ETV. Recurrent hydrocephalus or rescue diversion occurred in 5/227 patients (2.2%) after VP shunting, 34/140 (24.3%) after upfront resection, and 5/53 (9.4%) after ETV. In matched ETV versus VP-shunt pairs, infection occurred in 5/50 ETV patients (10.0%) versus 12/50 VP-shunt patients (24.0%; matched odds ratio [OR], 0.36; 95% confidence interval [CI], 0.12-1.14; P = 0.118), and recurrent hydrocephalus occurred in 5/50 ETV patients (10.0%) versus 4/50 VP-shunt patients (8.0%; matched OR, 1.25; 95% CI, 0.34-4.66; P = 1.000). The internally selected L2 logistic VP-shunt infection model achieved repeated cross-validation area under the receiver operating characteristic curve (AUC) 0.627 and Brier score 0.175. ETV recurrence was not modeled because only five events occurred.

**Discussion:**

The pathway comparisons are exploratory observational associations rather than causal effects. Both matched ETV comparisons were non-significant, with wide confidence intervals compatible with clinically opposite directions and important residual imbalance. The internally evaluated VP-shunt infection model is not independently validated and must not be used for individual treatment decisions. Larger prospective, anatomy-enriched, multi-institutional studies with complete follow-up are required.

## Introduction

Hydrocephalus is one of the most frequent and consequential presenting problems in children with posterior fossa tumors. In medulloblastoma, obstructive hydrocephalus often coexists with symptoms of raised intracranial pressure, urgent surgical planning, and the need to preserve a timely pathway to craniospinal irradiation and systemic therapy (1–5). The initial neurosurgical decision therefore has two parallel goals: to control intracranial pressure safely and to avoid complications that may interrupt oncological treatment.

Contemporary practice commonly includes three initial pathways. A preoperative VP shunt provides durable CSF diversion before tumor resection, but it also places permanent hardware into a patient who will soon undergo major posterior fossa surgery and oncological treatment (6–8). Upfront tumor resection avoids permanent preoperative diversion and may resolve obstruction when the fourth ventricle outflow pathway is restored, but persistent or recurrent hydrocephalus can still require rescue diversion (1–4). ETV offers an internal bypass without shunt hardware and is appealing when aqueductal or fourth ventricular outlet obstruction is dominant, but it is anatomically conditional and may fail early in selected children (9–13).

These competing risks make binary comparisons incomplete. A lower recurrence rate after shunting may be clinically meaningful only if weighed against infection risk, while the avoidance of shunt hardware with ETV must be weighed against the possibility of recurrent hydrocephalus. In retrospective data, this comparison is further complicated by confounding by indication: children selected for one pathway may differ systematically in age, ventricular size, tumor burden, dissemination, fontanelle status, and unrecorded anatomic features.

We therefore designed this study around two linked questions. First, we compared recorded postoperative infection and recurrent hydrocephalus across VP shunt, upfront resection, and ETV pathways using unadjusted descriptions and pairwise propensity score matching. Second, we conducted a transparent exploratory internal prediction analysis for postoperative infection after VP shunting. Because only five ETV recurrence events were observed, no ETV recurrence prediction model was fitted.

## Methods

### Study design, setting, and patients

This retrospective single-center cohort and prediction-model study was conducted at Beijing Tiantan Hospital. Eligible patients were younger than 18 years, had histologically confirmed medulloblastoma, had preoperative hydrocephalus documented before tumor-directed surgery, and had an identifiable initial hydrocephalus pathway. The cohort included consecutive patients treated from January 1, 2014, through March 1, 2018.

Patients were excluded if the diagnosis was not medulloblastoma, if hydrocephalus was absent before initial surgery, or if the hydrocephalus pathway could not be reconstructed. The study was approved by the institutional review board, and the requirement for individual informed consent was waived because the analysis used retrospective clinical data.

### Hydrocephalus pathways

Patients were classified according to the first definitive hydrocephalus strategy used before or at the start of tumor-directed treatment. The VP-shunt pathway included patients who underwent preoperative ventriculoperitoneal shunt placement followed by tumor resection. The upfront-resection pathway included patients who proceeded to tumor resection without permanent preoperative CSF diversion. The ETV pathway included patients who underwent preoperative endoscopic third ventriculostomy followed by tumor resection. Temporary drainage procedures used as emergency or bridging measures did not redefine the pathway unless they became the definitive strategy.

The pathway was chosen by the treating neurosurgical team rather than assigned by protocol. ETV selection was based on the treating neurosurgeon’s clinical and imaging assessment and experience. As a result, pathway comparisons were treated as observational contrasts. Propensity score matching was used to reduce measured baseline imbalance, but the analysis does not claim to reproduce a randomized comparison, particularly because detailed third-ventricular-floor and prepontine/interpeduncular cistern anatomy, obstruction site, acute deterioration, and surgeon-specific treatment preferences were not uniformly captured.

### Outcomes and predictors

The two clinical outcomes were postoperative intracranial infection and recurrent hydrocephalus or rescue CSF diversion. Each binary outcome represented any event documented during the available postoperative follow-up. Infection was defined from the recorded postoperative intracranial infection field and could include shunt infection, meningitis, ventriculitis, wound infection with intracranial extension, or culture-negative treated infection. Because source-level data did not consistently distinguish these components, the endpoint was analyzed as a non-decomposable composite and component frequencies could not be retrospectively reported. Recurrent hydrocephalus was defined as recurrent or persistent hydrocephalus requiring further CSF diversion or recorded as recurrence after the initial pathway. The minimum planned postoperative follow-up was 6 months, but exact individual ascertainment windows and dates of last contact, death, or loss to follow-up were unavailable. We therefore did not perform Kaplan-Meier or competing-risk analyses. A six-month sensitivity analysis used the recorded recurrence-time field only and did not treat it as complete follow-up time for the full cohort.

Baseline variables considered for matching included age, sex, pathological subgroup, Evans index, fontanelle status, and disseminated disease. Candidate VP-shunt infection predictors were restricted to the routinely recorded preoperative imaging fields used in the original analysis: Evans index, frontal horn diameter, and intracranial diameter. Numeric predictors were standardized within the modeling pipeline.

### Statistical analysis

Continuous variables are summarized as means with standard deviations or medians with interquartile ranges, as appropriate, and categorical variables as counts with percentages. Pairwise propensity score matching was performed for VP shunt versus upfront resection, ETV versus upfront resection, and ETV versus VP shunt. Propensity scores were estimated with logistic regression using age, sex, pathological subgroup, Evans index, fontanelle status, and disseminated disease, followed by 1:1 nearest-neighbor matching without replacement using a caliper of 0.2 standard deviations of the logit propensity score. Covariate balance was assessed with absolute standardized mean differences.

Outcome comparisons were reported in both unmatched and matched samples. Unmatched comparisons used Fisher exact odds ratios. Matched comparisons used discordant-pair odds ratios with exact McNemar P values, and matched event counts were shown to keep the direction and magnitude of each comparison visible. Six pathway-by-outcome comparisons were performed without multiplicity adjustment and were considered exploratory; emphasis was placed on effect estimates and 95% confidence intervals rather than dichotomous significance testing. This study was reported in accordance with the TRIPOD+AI and STROBE statements (16, 17).

### Prediction model development and internal evaluation

The VP-shunt infection model was developed only among the 227 patients in the VP-shunt pathway. Candidate approaches included penalized logistic regression with L1 or L2 regularization, class-weighted logistic regression, random over-sampling, random under-sampling, synthetic minority over-sampling technique (SMOTE), shallow decision trees, random forests, and gradient boosting. Selection was restricted to repeated stratified 5-fold cross-validation within this retrospective cohort. Imputation, standardization, and resampling were fitted only within each training fold to prevent information leakage.

Candidate configurations were fixed before reanalysis. The prespecified selection rule was highest mean cross-validation AUC; when candidates differed by less than 0.02 AUC, lower Brier score and then model simplicity resolved selection. Repeated cross-validation used random seeds 13, 29, 42, 77, and 101; tree-based estimators used random seed 42. Analyses used Python 3.9.6 and scikit-learn 1.6.1. No external or temporal evaluation is claimed.

Discrimination was summarized with the mean repeated cross-validation AUC and its distribution across folds, and overall probabilistic accuracy was summarized with the Brier score. Grouped calibration and standardized model coefficients are reported for transparency. ETV recurrence was not modeled because only five events occurred. Because the selected VP-shunt model was internally evaluated, showed limited discrimination, and has no clinical decision-support role, decision-curve analysis was not performed.

## Results

### Cohort assembly and pathway-specific endpoint burden

**Figure 1:**
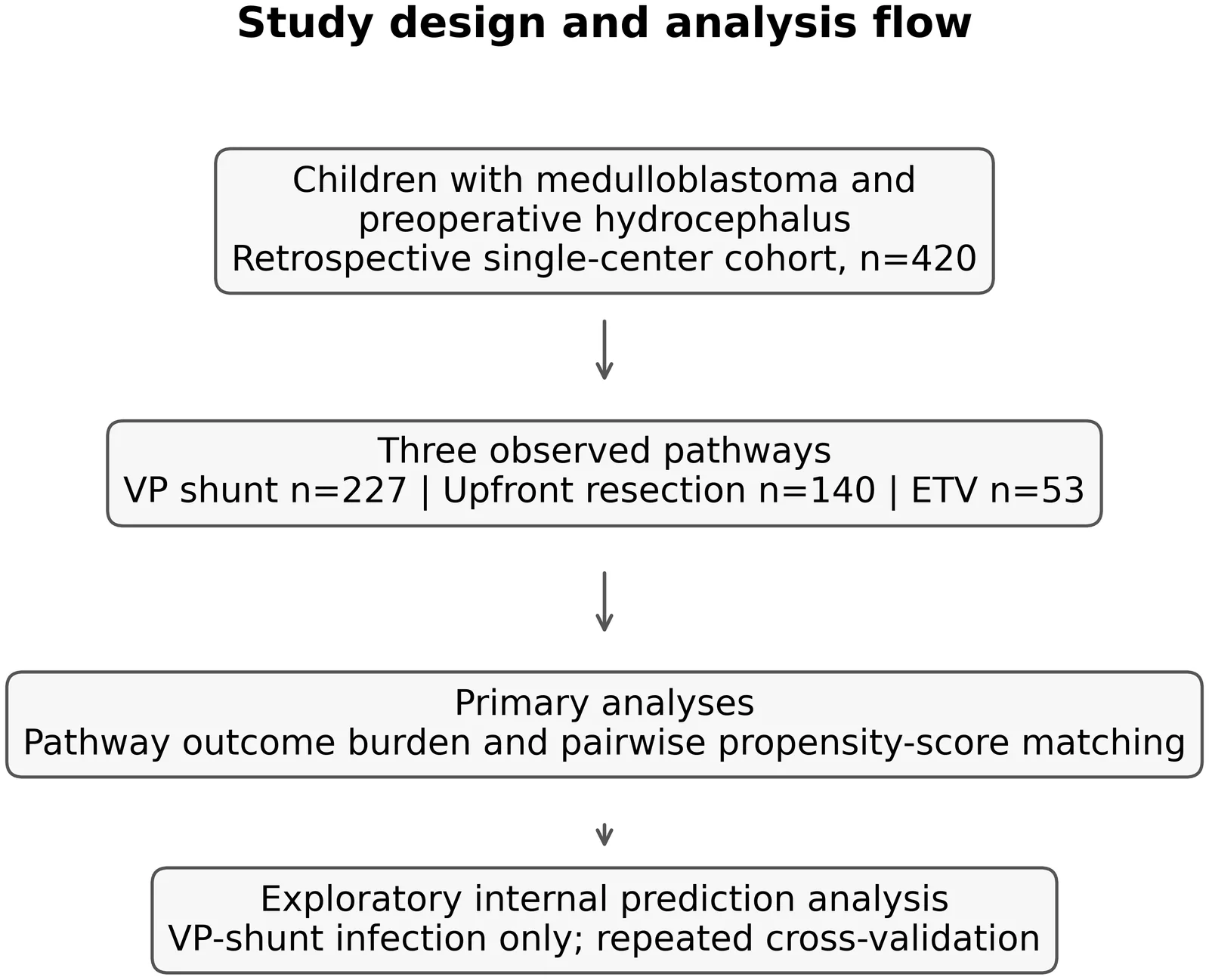
Study design and analysis flow. The image shows the 420-patient retrospective single-center cohort, the three hydrocephalus pathways, pairwise propensity score matching, and repeated cross-validation of the exploratory VP-shunt infection model.

The retrospective single-center cohort included 420 children with medulloblastoma-associated hydrocephalus treated from January 1, 2014, through March 1, 2018: 227 in the preoperative VP-shunt pathway, 140 in the upfront-resection pathway, and 53 in the ETV pathway. Baseline characteristics and endpoint rates by pathway are shown in **Table 1**, and the cohort and analysis flow is shown in **Figure 1**.

**Table 1:** Retrospective cohort characteristics and recorded binary outcomes.

Hydrocephalus pathway	No.	Age, median (IQR), y	Male sex	Evans index, median (IQR)	Open fontanelle	Postoperative intracranial infection	Hydrocephalus recurrence/rescue diversion
Preoperative VP shunt	227	7.0 (4.0-11.0)	143/227 (63.0%)	0.343 (0.306-0.394)	31/227 (13.7%)	55/227 (24.2%)	5/227 (2.2%)
Upfront resection only	140	6.0 (4.0-10.0)	85/140 (60.7%)	0.328 (0.303-0.384)	11/140 (7.9%)	31/140 (22.1%)	34/140 (24.3%)
Preoperative ETV	53	9.0 (6.0-11.0)	39/53 (73.6%)	0.328 (0.303-0.374)	4/53 (7.5%)	5/53 (9.4%)	5/53 (9.4%)

Values are counts with percentages unless otherwise specified. IQR, interquartile range; VP, ventriculoperitoneal; ETV, endoscopic third ventriculostomy.

**Figure 2:**
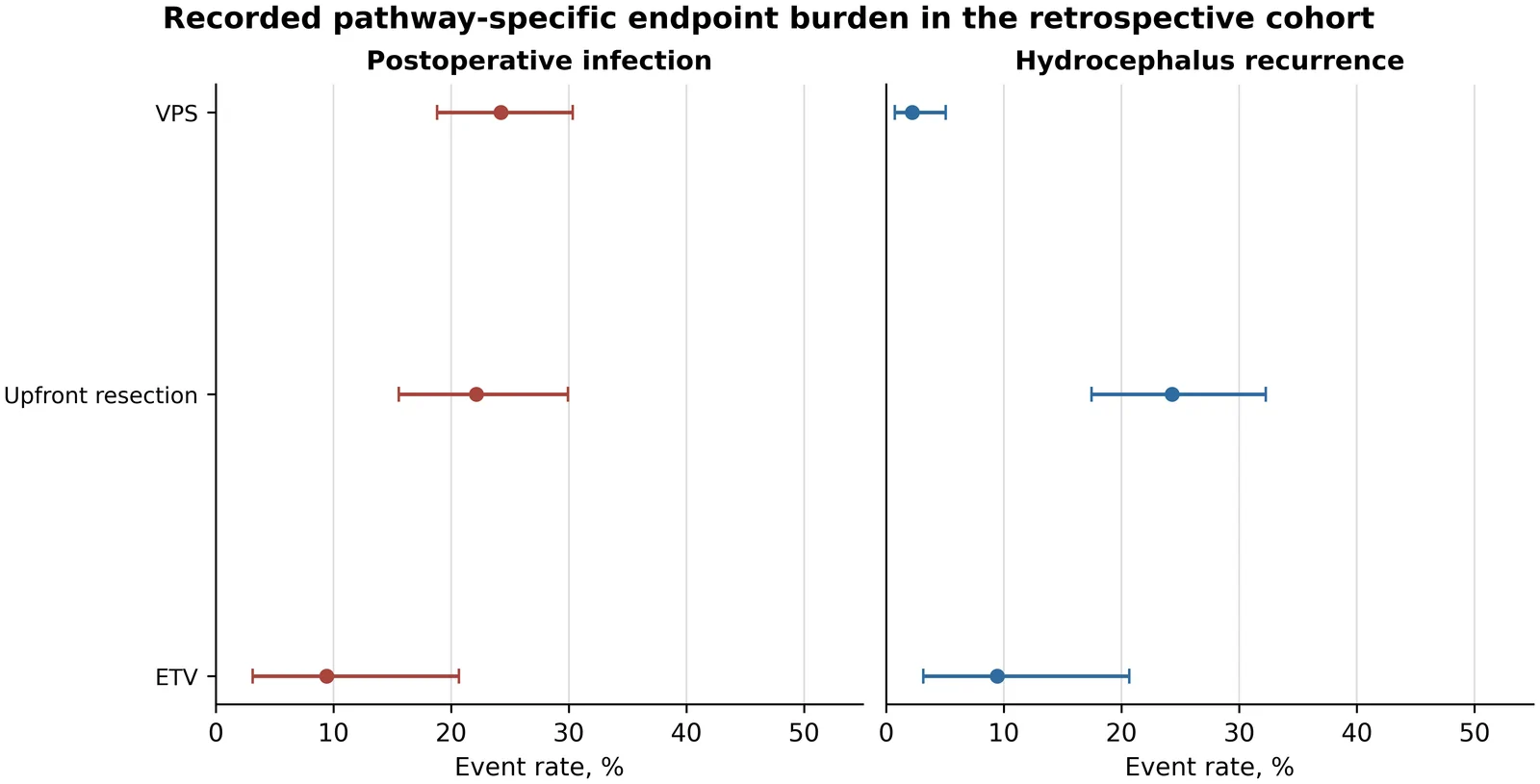
Recorded pathway-specific endpoint burden in the retrospective cohort. Points denote event rates and whiskers denote exact binomial 95% confidence intervals. All patients had a planned minimum postoperative follow-up of 6 months; individual censoring dates were unavailable.

Postoperative intracranial infection occurred in 55/227 (24.2%) after VP shunting, 31/140 (22.1%) after upfront resection, and 5/53 (9.4%) after ETV. Recurrent hydrocephalus or rescue diversion occurred in 5/227 (2.2%) after VP shunting, 34/140 (24.3%) after upfront resection, and 5/53 (9.4%) after ETV. **Figure 2** shows the absolute recorded endpoint burden with exact binomial confidence intervals.

### Pairwise propensity score matching across three pathways

Pairwise matching yielded 128 VP-shunt versus upfront-resection pairs, 46 ETV versus upfront-resection pairs, and 50 ETV versus VP-shunt pairs. The maximum residual standardized mean differences after matching were 0.169, 0.154, and 0.352, respectively. Balance improved across measured baseline variables, although the ETV versus VP-shunt comparison retained important residual imbalance (**Supplementary Table 1**; **Supplementary Figure 1**).

In matched VP-shunt versus upfront-resection pairs, the infection comparison showed VPS 33/128 (25.8%) vs Upfront resection 27/128 (21.1%); matched OR 1.30 (0.73-2.33), P = 0.461, whereas recurrent hydrocephalus was less frequent after VP shunting (VPS 4/128 (3.1%) vs Upfront resection 31/128 (24.2%); matched OR 0.07 (0.02-0.29), P<0.001). Matched ETV versus upfront-resection pairs showed ETV 3/46 (6.5%) vs Upfront resection 7/46 (15.2%); matched OR 0.33 (0.07-1.65), P = 0.289 for infection and ETV 5/46 (10.9%) vs Upfront resection 13/46 (28.3%); matched OR 0.38 (0.14-1.08), P = 0.096 for recurrence. In the matched ETV versus VP-shunt comparison, infection was lower after ETV (ETV 5/50 (10.0%) vs VPS 12/50 (24.0%); matched OR 0.36 (0.12-1.14), P = 0.118), whereas recurrent hydrocephalus was numerically higher after ETV (ETV 5/50 (10.0%) vs VPS 4/50 (8.0%); matched OR 1.25 (0.34-4.66), P = 1.000) Figure 5A; **Table 2**.

**Table 2:** Exploratory pairwise propensity score-matched observational contrasts across VP shunt, upfront resection, and ETV pathways.

Comparison	Outcome	Unmatched group A	Unmatched group B	Unmatched OR (95% CI)	Unmatched P	Matched group A	Matched group B	Matched OR (95% CI)	Matched P	Pairs	Discordant pairs
VP shunt vs upfront resection	Postoperative intracranial infection	VPS: 55/227 (24.2%)	Upfront resection: 31/140 (22.1%)	1.12 (0.68-1.86)	0.704	VPS: 33/128 (25.8%)	Upfront resection: 27/128 (21.1%)	1.30 (0.73-2.33)	0.461	128	b=26, c=20
VP shunt vs upfront resection	Hydrocephalus recurrence/rescue diversion	VPS: 5/227 (2.2%)	Upfront resection: 34/140 (24.3%)	0.07 (0.03-0.18)	<0.001	VPS: 4/128 (3.1%)	Upfront resection: 31/128 (24.2%)	0.07 (0.02-0.29)	<0.001	128	b=2, c=29
ETV vs upfront resection	Postoperative intracranial infection	ETV: 5/53 (9.4%)	Upfront resection: 31/140 (22.1%)	0.37 (0.13-1.00)	0.061	ETV: 3/46 (6.5%)	Upfront resection: 7/46 (15.2%)	0.33 (0.07-1.65)	0.289	46	b=2, c=6
ETV vs upfront resection	Hydrocephalus recurrence/rescue diversion	ETV: 5/53 (9.4%)	Upfront resection: 34/140 (24.3%)	0.32 (0.12-0.88)	0.026	ETV: 5/46 (10.9%)	Upfront resection: 13/46 (28.3%)	0.38 (0.14-1.08)	0.096	46	b=5, c=13
ETV vs VP shunt	Postoperative intracranial infection	ETV: 5/53 (9.4%)	VPS: 55/227 (24.2%)	0.33 (0.12-0.86)	0.016	ETV: 5/50 (10.0%)	VPS: 12/50 (24.0%)	0.36 (0.12-1.14)	0.118	50	b=4, c=11
ETV vs VP shunt	Hydrocephalus recurrence/rescue diversion	ETV: 5/53 (9.4%)	VPS: 5/227 (2.2%)	4.62 (1.29-16.61)	0.024	ETV: 5/50 (10.0%)	VPS: 4/50 (8.0%)	1.25 (0.34-4.66)	1.000	50	b=5, c=4

Group A is the first pathway named in each comparison. Matched estimates use discordant pairs. Six pathway-by-outcome comparisons were exploratory and no multiplicity adjustment was applied.

### Exploratory VP-shunt infection model

The VP-shunt infection model was developed in 227 patients with 55 infection events. All tested candidate strategies, feature sets, sampling methods, repeated cross-validation AUCs, Brier scores, and selection status are reported in **Supplementary Table 2**. Applying the prespecified selection rule, the internally selected model was L2 logistic regression without resampling.

Only five recurrent hydrocephalus or rescue-diversion events occurred among the 53 ETV patients. This event count was considered insufficient for credible prediction modeling, so the previous ETV recurrence model and all associated validation claims were removed. In the six-month sensitivity analysis, both matched ETV recurrence contrasts remained non-significant (**Supplementary Table 4**).

### Internal prediction performance

**Figure 3:**
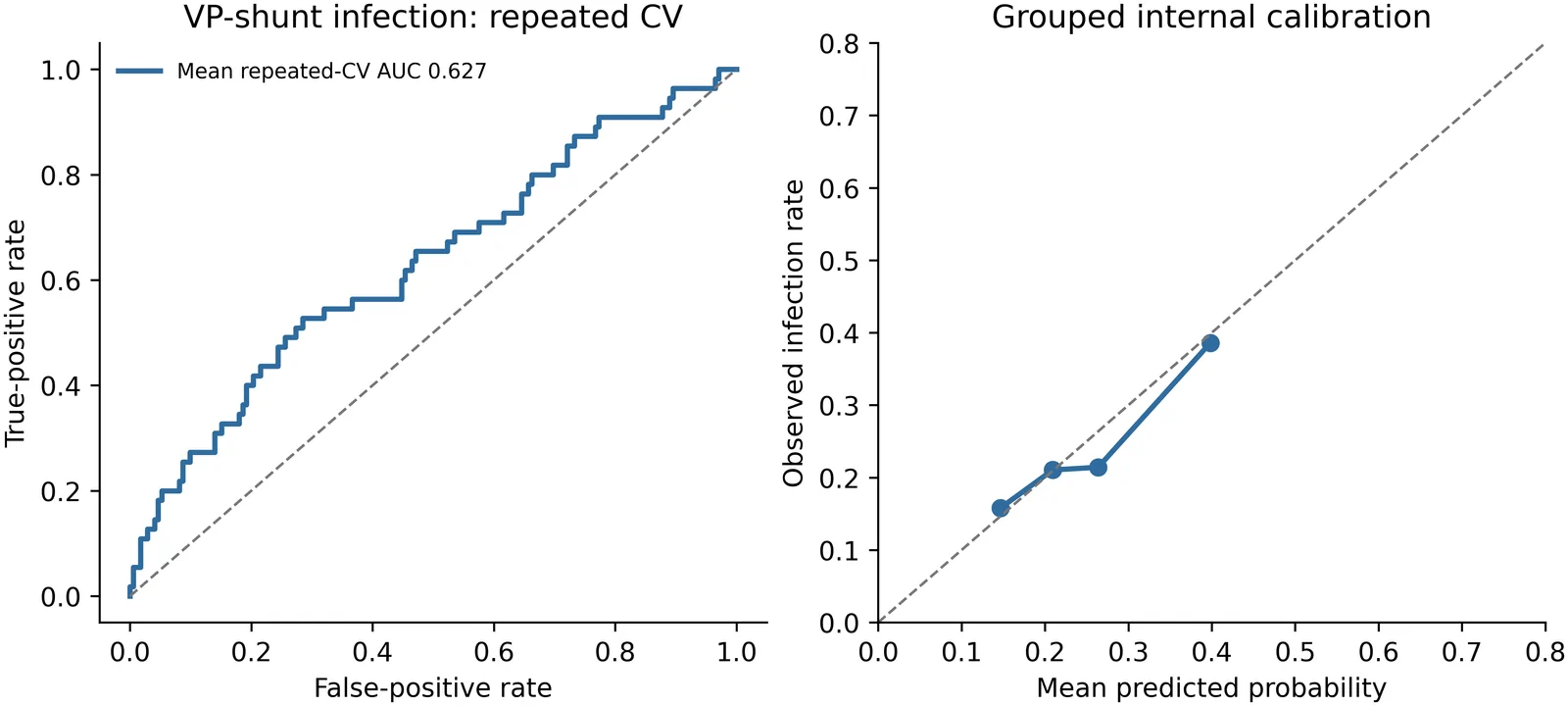
Exploratory internal VP-shunt infection prediction. Receiver operating characteristic and grouped calibration plots summarize repeated cross-validation performance. This is internal evaluation, not independent validation.

The internally selected VP-shunt infection model achieved mean repeated cross-validation AUC 0.627 and Brier score 0.175 (**Table 3**; **Figure 3**). The model retained three nonzero standardized coefficients: Evans index 0.287, frontal horn diameter 0.034, and intracranial diameter -0.533 (**Table 4**). These results represent exploratory internal evaluation only and do not establish independent validation or clinical utility.

**Table 3:** Exploratory internally evaluated VP-shunt infection model performance.

Model	Outcome	Model predictors	N/events	Repeated CV AUC (95% CI)	Repeated CV Brier
VP-shunt infection model (internally selected; exploratory)	Postoperative intracranial infection	evans_index, frontal_horn_diameter, intracranial_diameter	227/55	0.627 (0.537-0.717)	0.175

The internally selected L2 logistic model used Evans index, frontal horn diameter, and intracranial diameter. Performance is based on repeated stratified 5-fold cross-validation within the retrospective cohort; no external or temporal evaluation was performed.

**Table 4:** Standardized coefficients of the internally selected VP-shunt infection model.

Model	Predictor	Standardized coefficient	Odds ratio
VP-shunt infection model (internally selected; exploratory)	Evans index	0.287	1.332
VP-shunt infection model (internally selected; exploratory)	Frontal horn diameter	0.034	1.035
VP-shunt infection model (internally selected; exploratory)	Intracranial diameter	-0.533	0.587

Numeric variables were standardized inside each training fold. Coefficients are penalized conditional estimates and are not intended as unpenalized inference or mechanistic effects.

The negative conditional coefficient for intracranial diameter should not be interpreted mechanistically. In this small correlated imaging feature set, it may act as a proxy for age or head size and may be unstable. The complete candidate-model results and analysis settings are provided to support transparent reproduction rather than clinical implementation.

## Discussion

In this medulloblastoma-specific hydrocephalus study, three findings are most relevant. First, recorded complication patterns differed by pathway, but the comparisons were nonrandomized observational contrasts. Second, pairwise propensity score matching reduced measured imbalance, while the ETV-related estimates remained non-significant and the ETV versus VP-shunt comparison retained a maximum residual standardized mean difference of 0.352. Third, exploratory internal prediction of VP-shunt infection was possible, but discrimination was limited and no independent validation cohort was available.

The results should not be interpreted as a universal ranking of hydrocephalus strategies. VP shunting may provide durable control of CSF diversion but exposes the child to implanted hardware and infection risk before definitive tumor therapy. ETV avoids shunt hardware and may be attractive in obstructive hydrocephalus, but its feasibility and durability depend on anatomy that was only partially captured in the available data. Upfront resection remains reasonable in selected patients, but recurrent hydrocephalus or rescue diversion may still occur. The wide confidence intervals around the non-significant ETV estimates were compatible with clinically opposite directions.

The prediction analysis illustrates a common problem in pediatric neurosurgical datasets. Rare but clinically important endpoints encourage data-balancing or flexible algorithms, yet apparent gains can be unstable when event counts are small. For VP-shunt infection, all preprocessing and sampling were confined to training folds and model selection used repeated cross-validation only. For ETV recurrence, only five events were available; we therefore removed prediction modeling rather than present an unstable model.

VP shunts introduce hardware-related infection risks, whereas successful ETV depends on obstruction anatomy and an adequate CSF absorption pathway. Reports on complex pediatric hydrocephalus and staged treatment of critical neonatal hydrocephalus provide broader context for these mechanisms, although they are not direct evidence in medulloblastoma (14, 15). Future studies should prospectively capture third-ventricular-floor and prepontine/interpeduncular cistern anatomy, obstruction site, acute deterioration, operative details, surgeon experience, and treatment preference.

The internally evaluated VP-shunt model should not be used to select a hydrocephalus pathway or guide treatment for an individual child. Its AUC of 0.627 indicates limited discrimination, and the coefficients are reported for transparency rather than mechanistic inference. Decision-curve analysis was not added because there is no validated model or proposed clinical threshold for implementation.

## Limitations

Several limitations should be considered. First, this was a single-center retrospective study, and pathway selection was not randomized. Propensity score matching reduced measured imbalance but could not adjust for unmeasured selection factors, including detailed third-ventricular-floor and prepontine/interpeduncular cistern anatomy, obstruction site, acute clinical deterioration, surgeon experience, and treatment preference. Residual imbalance remained, particularly for ETV versus VP shunt. Second, postoperative intracranial infection could not be consistently subclassified into shunt infection, meningitis, ventriculitis, wound-related infection, or culture-negative treated infection. This non-decomposable composite limits mechanistic interpretation.

Third, exact individual outcome-ascertainment windows and dates of last contact, death, or loss to follow-up were unavailable. Although the minimum planned postoperative follow-up was 6 months and a six-month recurrence sensitivity analysis was performed, binary outcomes may still be affected by differences in follow-up; Kaplan-Meier and competing-risk analyses were not possible. Fourth, the VP-shunt infection model has internal repeated cross-validation results only and no independent evaluation cohort. Fifth, only five ETV recurrence events precluded credible prediction modeling. These limitations prevent causal, action-guiding, or generalizability claims.

## Conclusions

In children with medulloblastoma-associated hydrocephalus, VP shunt, upfront resection, and ETV showed different recorded postoperative outcome patterns, but these nonrandomized comparisons are exploratory associations rather than causal effects. The internally evaluated VP-shunt infection model is not validated for clinical use, and ETV recurrence was not modeled because of rare events. Prospective, anatomy-enriched, multi-institutional studies with complete follow-up are needed before prediction or pathway recommendations are considered.

## Data Availability

Deidentified data may be made available from the corresponding author on reasonable request, subject to institutional approval, patient-privacy protections, and applicable ethics restrictions.

## Supplementary material

Supplementary Figure 1 Absolute standardized mean differences before and after each pairwise propensity score match.
