# Supplementary material for: Hydrocephalus management pathways in pediatric medulloblastoma: a retrospective cohort study
Source: Front Oncol. 2026 Sep 8;16:1928797. doi: 10.3389/fonc.2026.1928797 (PMC13593456; doi:10.3389/fonc.2026.1928797)
Supplement: Supplementary file 1 [file Table1.docx]

Supplementary Table S1. Covariate balance before and after matching.

Abbreviations: AUC, area under the receiver-operating-characteristic curve; ETV, endoscopic third ventriculostomy; OR, odds ratio; PSM, propensity-score matching; VP, ventriculoperitoneal.

| **Comparison** | **Variable** | **SMD before** | **SMD after** |
| --- | --- | --- | --- |
| VP shunt vs upfront resection | age | 0.094 | 0.117 |
| VP shunt vs upfront resection | sex: Female | 0.047 | 0.064 |
| VP shunt vs upfront resection | sex: Male | 0.047 | 0.064 |
| VP shunt vs upfront resection | pathology: Non-WNT/SHH | 0.041 | 0.05 |
| VP shunt vs upfront resection | pathology: SHH-TP53MU | 0.33 | 0.06 |
| VP shunt vs upfront resection | pathology: SHH-TP53WT | 0.048 | 0.0 |
| VP shunt vs upfront resection | pathology: WNT | 0.157 | 0.091 |
| VP shunt vs upfront resection | evans_index | 0.113 | 0.0 |
| VP shunt vs upfront resection | fontanelle_status: Closed | 0.187 | 0.0 |
| VP shunt vs upfront resection | fontanelle_status: Open | 0.187 | 0.169 |
| VP shunt vs upfront resection | disseminated: No | 0.083 | 0.169 |
| ETV vs upfront resection | age | 0.413 | 0.116 |
| ETV vs upfront resection | sex: Female | 0.274 | 0.0 |
| ETV vs upfront resection | sex: Male | 0.274 | 0.0 |
| ETV vs upfront resection | pathology: Non-WNT/SHH | 0.172 | 0.045 |
| ETV vs upfront resection | pathology: SHH-TP53WT | 0.426 | 0.048 |
| ETV vs upfront resection | pathology: WNT | 0.344 | 0.0 |
| ETV vs upfront resection | evans_index | 0.077 | 0.154 |
| ETV vs upfront resection | fontanelle_status: Closed | 0.012 | 0.082 |
| ETV vs upfront resection | fontanelle_status: Open | 0.012 | 0.082 |
| ETV vs upfront resection | disseminated: No | 0.139 | 0.122 |
| ETV vs upfront resection | disseminated: Yes | 0.139 | 0.122 |
| ETV vs VP shunt | age | 0.318 | 0.141 |
| ETV vs VP shunt | sex: Female | 0.228 | 0.13 |
| ETV vs VP shunt | sex: Male | 0.228 | 0.13 |
| ETV vs VP shunt | pathology: Non-WNT/SHH | 0.213 | 0.201 |
| ETV vs VP shunt | pathology: SHH-TP53MU | 0.33 | 0.083 |
| ETV vs VP shunt | pathology: SHH-TP53WT | 0.472 | 0.21 |
| ETV vs VP shunt | pathology: WNT | 0.194 | 0.128 |
| ETV vs VP shunt | evans_index | 0.202 | 0.0 |
| ETV vs VP shunt | fontanelle_status: Closed | 0.198 | 0.0 |
| ETV vs VP shunt | fontanelle_status: Open | 0.198 | 0.352 |
| ETV vs VP shunt | disseminated: No | 0.057 | 0.352 |
