# Supplementary material for: Hydrocephalus management pathways in pediatric medulloblastoma: a retrospective cohort study
Source: Front Oncol. 2026 Sep 8;16:1928797. doi: 10.3389/fonc.2026.1928797 (PMC13593456; doi:10.3389/fonc.2026.1928797)
Supplement: Supplementary file 2 [file Table2.docx]

Supplementary Table S2. Internal candidate-model comparison for VP-shunt infection.

Abbreviations: AUC, area under the receiver-operating-characteristic curve; ETV, endoscopic third ventriculostomy; OR, odds ratio; PSM, propensity-score matching; VP, ventriculoperitoneal.

| **Outcome** | **Feature set** | **Algorithm** | **Sampling** | **Internal repeated CV AUC** | **Internal repeated CV Brier** | **Selection status** |
| --- | --- | --- | --- | --- | --- | --- |
| VP-shunt postoperative infection | evans_index, frontal_horn_diameter, intracranial_diameter | L2 logistic | None | 0.627 | 0.175 | Selected by internal-only rule |
| VP-shunt postoperative infection | evans_index, frontal_horn_diameter, intracranial_diameter | L1 logistic | None | 0.617 | 0.177 | Not selected |
| VP-shunt postoperative infection | evans_index, frontal_horn_diameter, intracranial_diameter | L1 logistic, class-weighted | None | 0.619 | 0.243 | Not selected |
| VP-shunt postoperative infection | evans_index, frontal_horn_diameter, intracranial_diameter | L1 logistic | Random under-sampling | 0.61 | 0.245 | Not selected |
| VP-shunt postoperative infection | evans_index, frontal_horn_diameter, intracranial_diameter | L1 logistic | Random over-sampling | 0.623 | 0.24 | Not selected |
| VP-shunt postoperative infection | evans_index, frontal_horn_diameter, intracranial_diameter | L1 logistic | SMOTE (k=2 surrogate) | 0.622 | 0.241 | Not selected |
| VP-shunt postoperative infection | evans_index, frontal_horn_diameter, intracranial_diameter | Decision tree (depth 2) | None | 0.544 | 0.194 | Not selected |
| VP-shunt postoperative infection | evans_index, frontal_horn_diameter, intracranial_diameter | Random forest (depth 2) | None | 0.565 | 0.23 | Not selected |
| VP-shunt postoperative infection | evans_index, frontal_horn_diameter, intracranial_diameter | Gradient boosting (stumps) | None | 0.581 | 0.18 | Not selected |

Selection rule: highest mean repeated stratified 5-fold cross-validation AUC; when candidates were within 0.02 AUC, lower Brier score and then model simplicity were used. No later cohort was used.
