# Supplementary material for: Hydrocephalus management pathways in pediatric medulloblastoma: a retrospective cohort study
Source: Front Oncol. 2026 Sep 8;16:1928797. doi: 10.3389/fonc.2026.1928797 (PMC13593456; doi:10.3389/fonc.2026.1928797)
Supplement: Supplementary file 3 [file Table3.docx]

Supplementary Table S3. Descriptive timing among patients with recorded recurrence events.

Abbreviations: AUC, area under the receiver-operating-characteristic curve; ETV, endoscopic third ventriculostomy; OR, odds ratio; PSM, propensity-score matching; VP, ventriculoperitoneal.

| **Pathway** | **Events with recorded timing** | **Median days to recurrence** | **IQR, days** | **Range, days** | **Interpretation** |
| --- | --- | --- | --- | --- | --- |
| Preoperative VP shunt | 5 | 57.0 | 30.0-70.0 | 28-88 | Event-time descriptor only; not cohort follow-up |
| Upfront resection only | 34 | 41.5 | 23.2-77.5 | 7-710 | Event-time descriptor only; not cohort follow-up |
| Preoperative ETV | 5 | 40.0 | 30.0-100.0 | 14-120 | Event-time descriptor only; not cohort follow-up |

Recorded event timing is not a measure of follow-up for the full cohort and was not used for survival analysis.
