# Supplementary material for: Hydrocephalus management pathways in pediatric medulloblastoma: a retrospective cohort study
Source: Front Oncol. 2026 Sep 8;16:1928797. doi: 10.3389/fonc.2026.1928797 (PMC13593456; doi:10.3389/fonc.2026.1928797)
Supplement: Supplementary file 4 [file Table4.docx]

Supplementary Table S4. Six-month recurrence/rescue-diversion sensitivity analysis.

Abbreviations: AUC, area under the receiver-operating-characteristic curve; ETV, endoscopic third ventriculostomy; OR, odds ratio; PSM, propensity-score matching; VP, ventriculoperitoneal.

| **Comparison** | **Outcome** | **Unmatched group A** | **Unmatched group B** | **Unmatched OR (95% CI)** | **Unmatched P** | **Matched group A** | **Matched group B** | **Matched OR (95% CI)** | **Matched P** | **Pairs** | **Discordant pairs** |
| --- | --- | --- | --- | --- | --- | --- | --- | --- | --- | --- | --- |
| VP shunt vs upfront resection | Hydrocephalus recurrence/rescue diversion within 6 months | VPS: 5/227 (2.2%) | Upfront resection: 28/140 (20.0%) | 0.09 (0.03-0.24) | <0.001 | VPS: 4/128 (3.1%) | Upfront resection: 25/128 (19.5%) | 0.09 (0.02-0.37) | <0.001 | 128 | b=2, c=23 |
| ETV vs upfront resection | Hydrocephalus recurrence/rescue diversion within 6 months | ETV: 5/53 (9.4%) | Upfront resection: 28/140 (20.0%) | 0.42 (0.15-1.14) | 0.090 | ETV: 5/46 (10.9%) | Upfront resection: 9/46 (19.6%) | 0.56 (0.19-1.66) | 0.424 | 46 | b=5, c=9 |
| ETV vs VP shunt | Hydrocephalus recurrence/rescue diversion within 6 months | ETV: 5/53 (9.4%) | VPS: 5/227 (2.2%) | 4.62 (1.29-16.61) | 0.024 | ETV: 5/50 (10.0%) | VPS: 4/50 (8.0%) | 1.25 (0.34-4.66) | 1.000 | 50 | b=5, c=4 |

This sensitivity analysis restricts recurrence/rescue diversion to events recorded within 180 days. Matching was re-derived using the same baseline covariates as in the primary analysis.
